# Response to Should sleeve gastrectomy be a preoperative standard in kidney transplant waitlisted patients with a BMI of 35 kg/m^2^

**DOI:** 10.1007/s11695-022-06199-2

**Published:** 2022-07-11

**Authors:** Tomasz Dziodzio, Johann Pratschke, Robert Öllinger

**Affiliations:** 1 Department of Surgery–Campus Charité Mitte and Campus Virchow-Klinikum, Charité–Universitätsmedizin Berlin, Augustenburger Platz 1, 13352 Berlin, Germany; 2 BIH Charité Clinician Scientist Program, Berlin Institute of Health (BIH), Berlin, Germany

Response

We thank the Polish colleagues for their thoughtful letter and their contribution to the discussion of our publication. We absolutely agree that the body mass index (BMI) is an imperfect method of measuring the severity of obesity in patients with chronic kidney disease. First, it is less accurate than abdominal adiposity, waist circumference, and waist-to-hip ratio measurements for assessing obesity-related diseases and complications [1, 2]. Second, obesity assessment using the BMI neglects the patient’s body composition and the proportion between (abdominal) fat, fluid, and muscle tissue. While the former is associated with the metabolic syndrome, the latter seems to have a protective effect in dialysis patients and might contribute to the so-called obesity paradox in these patients [3, 4]. However, it is important to note that the data supporting the effects of the obesity paradox are mainly based on BMI measurements. Publications that are more recent question the existence obesity paradox and rather suggest that the hypothesized effects are biased by the imperfection of BMI measurement itself [5, 6]. On the other hand, BMI is a quick and simple tool and can be easily performed by any medical or non-medical personnel. Therefore, there is still a raison d'être for the BMI as a screening tool to identify patients at risk. However, patient identification should be followed by more elaborate obesity measures to qualify patients for further obesity treatment. The development of chronic kidney disease is a lengthy process: years pass between the onset of kidney disease, the need for dialysis, and kidney transplantation. Therefore, it is particularly important to identify potential patients at an early stage of their disease and offer obesity treatment as early as possible to reduce long-term complications, facilitate access to transplant programs, and improve patient survival and transplant outcomes. It is essential that such programs involve a multidisciplinary team of nurses, physical therapists, dietitians, internists, and bariatric and transplant surgeons. As bariatric surgery remains the only sustainable treatment option for morbid obesity, we believe kidney transplant candidates with a BMI of ≥ 35 kg/m^2^ should be evaluated for obesity treatment involving bariatric surgery early in the disease. Additionally, prehabilitation and ERAS (enhanced recovery after surgery) programs are becoming more important not only in bariatric surgery but also in kidney transplant candidates and should be implemented in the preparation process to achieve sustainable outcomes [7, 8]. In the coming years, the rate of marginal organs will remain high, and the scarcity of donor organs will prevail. Therefore, a relevant impact on transplant outcomes can be achieved mainly by influencing modifiable factors such as obesity in the recipient. We strongly believe obesity surgery programs bear the potential to overcome the weight stigma in selected kidney transplant candidates. However, it is crucial that such programs undergo prospective, high-quality scientific monitoring and evaluation to demonstrate their true clinical value and evidence in this patient population.
